# Clarifying Deeper Psychological Characteristics of Hikikomori Using the Rorschach Comprehensive System: A Pilot Case–Control Study

**DOI:** 10.3389/fpsyt.2019.00412

**Published:** 2019-06-13

**Authors:** Ryoko Katsuki, Ayako Inoue, Sílvia Indias, Keita Kurahara, Nobuki Kuwano, Fumika Funatsu, Hiroaki Kubo, Shigenobu Kanba, Takahiro A. Kato

**Affiliations:** ^1^Department of Neuropsychiatry, Graduate School of Medical Sciences, Kyushu University, Fukuoka, Japan; ^2^Department of Social Psychology, University of the Basque Country (UPV-EHU), San Sebastián, Spain; ^3^Center for Health Science and Counseling, Kyushu University, Fukuoka, Japan

**Keywords:** social withdrawal (hikikomori), the Rorschach Comprehensive System, adolescence, passive aggressive personality, dependence, amae, assertion

## Abstract

Hikikomori, a form of severe social withdrawal more than 6 months, has increasingly become a crucial issue especially among adolescents. Loneliness, avoidant personality, Japanese culture-related attachment style (“amae”), and difficulty in expressing emotions are suggested to be related to hikikomori. However, deeper psychological aspects have not been well clarified. The Rorschach test is one of the most popular psychological assessment tools to evaluate deeper personality traits. The Rorschach Comprehensive System (CS) has been established as the most reliable scoring method. Until now, no CS research has been conducted focusing on hikikomori. Therefore, we herein conducted a pilot case–control study using CS in clinical cases with and without hikikomori condition. Participants were recruited from the Mood Disorder/Hikikomori Clinic at Kyushu University Hospital. Twenty-two patients with hikikomori (HK patients) and 18 patients without hikikomori (non-HK patients) participated in the present study. All the 40 participants conducted the self-report Structured Clinical Interview for *DSM-IV* Axis II Personality Disorders (SCID-II) personality questionnaire and CS. Regarding the SCID-II personality questionnaire, various personality traits including passive aggressive trait were significantly higher in HK patients. Among CS variables, HK patients showed higher scores on FC (Form Color) and SumT (total number of texture-related responses). In addition, frequency of SumT was higher in HK patients. The present results suggest that persons with hikikomori are more likely to express emotions indirectly and expect others to presume their feelings and thoughts. Persons with hikikomori may also have difficulty in becoming independent emotionally from primitive dependence and attachment on significant others. Further investigations with larger samples are warranted for validation.

## Introduction

Hikikomori, a form of severe and prolonged social withdrawal, began to garner attention in Japan especially after the publication of *Hikikomori: Adolescence Without End* by a psychiatrist/psychopathologist, T. Saito, in 1998 (1). Hikikomori is now regarded as a crucial sociocultural issue especially among adolescents (1–3). Our previous case study has suggested the presence of persons with hikikomori who have adolescent mentalities even after the age of adolescence (4). Therefore, even though persons with hikikomori may be past the age of so-called adolescence, it is important to consider their problems as adolescent issue. We have previously been defined hikikomori as a state of social withdrawal combined with avoidance of major social interactions and responsibilities (e.g., education, employment, and friendship) lasting at least 6 months (5). Previous research has revealed that the majority of cases with hikikomori condition are comorbid with psychiatric disorders. A variety of hikikomori-related case reports and clinical studies have shown the possible relationships between psychological aspects, personality traits, and hikikomori (6). We have revealed that loneliness and avoidant personality are strongly linked to hikikomori (7). In addition, previous studies have suggested psychological and behavioral features of hikikomori such as Japanese culture-related attachment style (“amae”) and difficulty in expressing emotions (8); however, deeper psychological and personality aspects have not been well clarified.

The Rorschach test is one of the most well-known psychological assessment tools to evaluate broad and deeper personality traits such as personality organization and functioning including unconscious defense mechanisms. This tool can reveal deeper psychological aspects in various psychiatric disorders (9–11). Currently, the Rorschach Comprehensive System (hereafter referred to as CS) has been established as the most popular scoring method in terms of its standardized administration rules, high inter-rater and test–retest reliability, and high statistical construct validity (12, 13). CS has revealed various deeper psychological aspects in psychosomatic and psychiatric disorders including alexithymia and chronic pain (14, 15). However, to our knowledge, no CS research has been conducted focusing on hikikomori. We believe that CS can evaluate deeper psychological mechanisms that are linked to the roots of hikikomori such as loneliness, avoidant personality, “amae,” and difficulty in expressing emotions. Therefore, as a pilot study to grasp deeper hikikomori-related psychological and personality traits, we herein conducted a case–control study using CS in psychiatric patients with and without hikikomori condition.

## Methods

This study was performed in accordance with the Declaration of Helsinki and was approved by the ethics committees of Kyushu University, Fukuoka, Japan.

The Department of Neuropsychiatry at Kyushu University Hospital has recently established a Mood Disorder/Hikikomori Clinic (16, 17), where hikikomori condition is evaluated based on the semi-structured diagnostic interview (5). In the present study, research staff recruited clinical cases from the Mood Disorder/Hikikomori Clinic, from June 2014 to March 2016. Patients were informed of the aims and methods regarding the present study and that their participation was completely voluntary. Patients who agreed to participate in the present study then registered as study participants with written informed consent. The participants received a gift card incentive worth approximately $18.

### Self-Report SCID-II Personality Questionnaire

The Structured Clinical Interview for *DSM-IV* Axis II Personality Disorders (SCID-II) is a semi-structured interview for assessment of Axis II 10 personality disorders based on the *Diagnostic and Statistical Manual of Mental Disorders*, 4th edition (*DSM-IV*), such as avoidant personality disorder, depressive personality disorder, and passive aggressive personality disorder (18). All the participants conducted a self-rated screening personality questionnaire, the self-report SCID-II personality questionnaire, which can estimate 10 personality traits with 119 items by a 2-point scale of yes (1) and no (0).

### The Rorschach Comprehensive System

As shown the above, the CS has been established as the most reliable scoring method (12, 13). We referred to Takahashi et al. for scoring form quality based on standardized data from the Japanese population (19). CS considers a normal protocol test with less than 14 responses as invalid, and no participant was eliminated from the analysis based on this criterion. A clinical psychologist (AI) administered CS and scored the responses of all the participants. The tentative scores were reviewed by the examiner (AI) and another clinical psychologist (RK), and finalized after discrepancies had been discussed in order to strengthen the reliability of the present CS data.

### Statistical Analysis

Student’s *t*-test was conducted for comparison of SCID-II subscales between the HK patients and non-HK patients. For comparison on the Rorschach CS variables, medians of major variables were compared between HK patients and non-HK patients using the Mann–Whitney *U* test, and frequency data for CS major variables were examined using the chi-squared test. All analyses were performed using IBM SPSS 24 Advanced Statistics for Mac OS. For all analyses, a probability of value of *p* < 0.05 was considered significant.

## Results

We recruited 22 clinical cases (male = 12, female = 10, age *mean* = 33.14, SD = 9.33) who met the definition of hikikomori (a 6-month or longer period of time spent mostly at home; hereafter referred to as “HK patients”) and 18 clinical cases (male = 11, female = 7, age *mean* = 37.94, SD = 8.93) without hikikomori condition in their present and previous history (as “non-HK patients”). All the cases agreed to participate in the study, and a total of 40 clinical participants enrolled in the following assessments and comparison analysis.

The average age of onset among 22 HK patients was 21.67 years (SD = 7.64). The average period of withdrawal at present was 5.25 years (SD = 4.10). In addition, 11 cases (50%) of the HK patients had previous experience of hikikomori. Many hikikomori sufferers are known to begin to withdraw from society at their adolescence; thus, we believe that analysis of the data using the present HK patients provides us valuable knowledge in the understanding of the psychopathology of hikikomori with adolescent problems. Regarding home environment, 20 cases (91%) of HK patients and 11 cases (61%) of non-HK patients lived with others (mainly family members).

All the participants received a psychiatric diagnosis based on the *DSM-IV* (20). In HK patients, primary psychiatric diagnoses were, in order, the minor depressive disorder (*n* = 17), major depressive disorder (*n* = 3), social anxiety disorder (*n* = 1), and psychosis (*n* = 1). Regarding non-HK patients, primary psychiatric diagnoses were, in order, minor depressive disorder (*n* = 10), major depressive disorder (*n* = 3), panic disorder (*n* = 1), social anxiety disorder (*n* = 1), bipolar affective disorder (*n* = 1), psychosis (*n* = 1), and alcohol use disorder (*n* = 1) (**Table 1**).

**Table 1:** Primary psychiatric diagnoses on patients with hikikomori (HK patients) and patients without hikikomori (non-HK patients).

Diagnosis	HK patients (*N* = 22)		Non-HK patients (*N* = 18)	
	Number	%	Number	%
Major depressive disorder	3	13.6	3	16.7
Minor depressive disorder	17	77.3	10	55.6
Bipolar affective disorder	0	0	1	5.6
Psychosis	1	4.5	1	5.6
Social anxiety disorder	1	4.5	1	5.6
Panic disorder	0	0	1	5.6
Alcohol use disorder	0	0	1	5.6

Significant differences were found in various personality traits between HK patients and non-HK patients. Total scores of personality traits of avoidant, depressive, narcissistic, paranoid, passive aggressive, schizoid, and schizotypal personality in the SCID-II personality questionnaire were significantly higher in HK patients compared with non-HK patients (**Table 2**).

**Table 2:** Comparisons of Structured Clinical Interview for *DSM-IV* Axis II Personality Disorders (SCID-II) personality questionnaire between HK patients and non-HK patients.

Variables	HK patients (*N* = 22)		Non-HK patients (*N* = 18)		Statistics	
	Mean	SD	Mean	SD	t	p-value
**SCID-II**						
Avoidant personality	4.67	1.56	3.17	1.95	−2.67	0.011*
Independent personality	3.14	1.93	2.44	2.18	−1.064	0.294
Obsessive-compulsive personality	4.43	1.99	3.44	2.04	−1.524	0.136
Passive aggressive personality	2.68	1.76	1.17	1.58	−2.837	0.007*
Depressive personality^#^	5.05	1.80	3.33	2.57	−2.44	0.02*
Paranoid personality	3.57	1.99	1.41	1.50	−3.699	0.001**
Schizotypal personality	3.62	2.62	1.94	2.15	−2.158	0.037*
Schizoid personality^#^	3.67	1.28	2.50	1.72	−2.368	0.022*
Histrionic personality	1.00	1.20	1.00	1.28	0	1
Narcissistic personality^#^	3.68	2.73	1.83	1.89	−2.522	0.02*
Borderline personality	4.82	3.05	3.67	3.50	−1.112	0.273
Antisocial personality^#^	1.41	1.82	0.61	0.70	−1.757	0.087

Statistical p-values were derived from Student’s t-test. *p < 0.05, **p < 0.01.

^#^There were missing values.

CS analysis has revealed some interesting outcomes in hikikomori. Mann–Whitney *U* test showed that the score of FC (Form Color) in the cluster of “affect” was significantly higher in HK patients compared with non-HK patients. The score of SumT (sum of the number of texture-related responses) in the cluster of “interpersonal perception and behavior” was also higher in HK patients compared with non-HK patients (**Table 3**). In addition, as our purpose in the present study was an exploratory pilot study with small samples, we newly compared frequency of SumT on two conditions: SumT = 0 and SumT ≥ 1. The frequency of SumT ≥ 1 in HK patients was significantly higher (40.9%) than in non-HK patients (11.1%; *p* < 0.05). Among 22 HK patients, nine cases showed one or two texture-related responses, including two cases that showed two texture-related responses. On the other hand, among 18 non-HK patients, only two cases showed one texture-related response and 16 cases showed no texture-related response (**Table 4**). Food response (Fd) is also one of the variables in “interpersonal perception and behavior.” There was no significant difference on the score of Fd between HK patients and non-HK patients (**Table 3**). The results of comparisons on other CS variables are shown in **Supplementary Table 1**.

**Table 3:** Comparisons of Rorschach Comprehensive System (CS) variables between HK patients and non-HK patients.

Cluster	Variable name	HK patients (*N* = 22)			Non-HK patients (*N* = 18)			U	Z	p-value
		Mean	Median	SD	Mean	Median	SD			
Affect	FC	2.50	2.50	1.68	1.39	2.00	1.14	123	−2.1	0.037*
	CF + C	2.50	2.00	2.43	2.17	2.00	1.92	183	−0.4	0.68
	pureC	0.41	0.00	0.96	0.50	0.00	1.04	182	−0.6	0.55
	SumC'	1.55	1.00	1.77	1.67	1.00	1.33	168.5	−0.8	0.41
	WSumC	3.95	3.75	2.89	3.11	3.00	2.33	161	−1.0	0.31
	Afr	0.51	0.46	0.16	0.47	0.40	0.21	162	−1.0	0.33
	S	3.18	3.00	2.44	2.89	3.00	1.71	194	−0.1	0.91
	Blends	3.14	2.50	2.32	2.61	2.50	1.54	183	−0.4	0.68
	R	24.91	23.50	8.48	24.67	22.50	6.21	191.5	−0.2	0.86
	CP	0.00	0.00	0.00	0.00	0.00	0.00	198	0	1
Interpersonal perception and behavior	COP	0.27	0.00	0.55	0.61	0.00	0.92	161.5	−1.2	0.29
	AG	0.45	0.00	1.14	0.44	0.00	0.51	163	−1.2	0.25
	GHR	2.91	3.00	1.97	4.33	3.00	3.24	149	−1.4	0.18
	PHR	2.73	3.00	1.80	1.83	2.00	0.99	138	−1.7	0.09
	a	2.64	2.00	2.82	3.50	2.50	3.38	170	−0.8	0.44
	p	4.32	4.00	2.59	3.78	4.00	2.10	180	−0.5	0.62
	Food	0.82	1.00	0.85	1.11	1.00	0.76	154	−1.3	0.19
	SumT	0.50	0.00	0.67	0.11	0.00	0.32	137	−2.1	0.033*
	Human content	5.73	6.00	2.60	5.94	4.50	3.73	192.5	−0.2	0.88
	pureH	2.09	2.00	1.51	2.50	2.00	2.60	195.5	−0.1	0.95
	PER	1.09	0.00	2.41	1.06	0.50	1.73	182	−0.5	0.63
	Isolation	0.13	0.12	0.10	0.14	0.12	0.10	189.5	−0.3	0.82

Statistical p-values were derived from Mann–Whitney U test. *p < 0.05, **p < 0.01.

FC, Form Color Response; CF+C, Color Form Response + Pure Color Response; SumC', Sum of Achromatic Color Determinants; WSumC, Weighted Sum of Chromatic Determinants; Afr, Affective Ratio; S, White Space Details; R, Response; CP, Color Projection; COP, Coorporative Movement; AG, Aggressive Movement; GHR, Good Human Representation; PHR, Poor Human Representation; a, active; p, passive; PER, Personalized answer.

**Table 4:** Frequency of texture-related responses (SumT = 0, SumT ≥ 1) among HK patients and non-HK patients.

Characteristic	HK patients (*N* = 22)		Non-HK patients (*N* = 18)	
	Number	%	Number	%
SumT = 0	13	59.1	16	88.9
SumT ≥ 1 (SumT = 1 and SumT = 2)	9	40.9	2	11.1

## Discussion

This is the first case–control study to reveal some deeper psychological and personality features for clinical cases with hikikomori condition using the scores of CS.

The SCID-II personality questionnaire showed a positive relationship between hikikomori and various personality traits including passive aggressive personality. Passive aggressive personality can be described as critical in social situations with extra-punitive expression (20). Persons with this personality tend to express anger and aggression in more indirect ways. The present result suggests that persons with hikikomori condition are more likely to express their aggressive feelings in more indirect ways.

FC in CS is known to indicate the tendency to adjust emotions to others and the environment, or the likeliness to suppress emotional expressions when a person is moved or shaken up by social situations (12, 21). Based on the standard textbook interpretation, persons with larger FC tend to control their feelings and to express emotions indirectly and/or implicitly (12). Larger FC is related to the above-mentioned high passive aggressiveness. Thus, our finding of larger FC in HK patients may explain the deeper psychological mechanism of high passive aggressiveness in hikikomori. Based on these outcomes, we propose that it is important to acquire the skill to express emotions more directly and effectively in terms of hikikomori treatment and prevention. Future intervention approaches such as assertive training should be developed and applied in the clinical practice and prevention of hikikomori.

Texture-related response in CS represents needs and openness to close emotional relationships. SumT, the total number of texture-related responses, was larger in HK patients compared with non-HK patients in the present study. Interestingly, the frequency of SumT ≥ 1 in HK patients was significantly higher (40.9%) than in non-HK patients (11.1%). According to the standard interpretation of CS, this result can be interpreted that persons with hikikomori condition tend to have affirmative view toward others and expect more close emotional relationships (21). However, this interpretation may be contradictory to the actual behaviors of social withdrawal. We suppose the contradictory may be related to sociocultural background. According to the CS database of Japanese normal adults (*n* = 400), SumT = 0 appears among 57% of Japanese healthy adults, SumT = 1 appears among 29%, and SumT = 2 appears among 14% (22). Considering Japanese sociocultural background, persons with SumT = 1 may have emotional relationship with others based on need for affection and try to maintain the relationship (22). On the other hand, Takahashi et al. reported that Japanese persons with SumT = 1 can also be interpreted that they are not satisfied with the present social relationship and thus are strongly seeking close emotional relationships (22). In the present study, the frequency of SumT ≥ 1 in HK patients was significantly higher (40.9%) than in non-HK patients (11.1%; *p* < 0.05). What is this result showing? Based on the above (22), we herein propose that strong feeling of loneliness in persons with hikikomori condition may lead to longing for close emotional relationships.

Another notable point is that texture-related responses in CS are known to be related to attachment, dependence, and psychological reliance especially based on experiences between a child and a caregiver (mainly mother) in childhood (12). Interestingly, [SumT > 1] is one of the criteria in the Coping Deficit Index (referred to as CDI) in CS (12). A person with high CDI is regarded to have difficulties in adjusting to social situations (12). In the present study, 91% of HK patients lived with others (mainly with parents). A survey by the Japan’s Cabinet Office showed that many persons with hikikomori condition are under protection of their family both physically and financially (23). Therefore, we propose that some persons, who have high desires for basic affection and emotional support, may tend to withdraw into their home when they cannot satisfy their needs for basic affection and emotional support in social situations such as school and the workplace.

On the other hand, food content (Fd) is another CS variable related to dependence and psychological reliance. If an adult shows Fd ≥ 1, in the standard textbook interpretation, the person tends to show more dependent behaviors than expected (22). In other words, persons with more Fd tend to expect others to be tolerant and to act for their demands. According to the CS database of Japanese normal adults (*n* = 400), Fd = 0 appears among 65% of Japanese healthy adults, Fd = 1 appears among 26%, and Fd ≥ 2 appears among only 9% (22). Interestingly, in the present study, 13 HK patients (59%) and 15 non-HK patients (83%) showed Fd ≥ 1, but no significant difference was found between HK patients (Fd mean = 0.82, Mdn = 1) and non-HK patients (Fd mean = 1.11, Mdn = 1). According to a previous study by Schefar [as cited in Ref. (12)], Fd is related to desire for oral-phase dependence (12). Therefore, Fd response may indicate an immature and undifferentiated desire for dependence and one-sided dedication. On the other hand, texture-related response represents sense of touch such as a silk-soft feel or wrinkled hand, which needs self-others differentiation (24). Texture-related responses are produced based on experience of touching/being touched (12). Therefore, persons with texture-related response may have an affirmative impression of touching and ask for relationship with others.

Both HK patients and non-HK patients may have immature and undifferentiated desire for dependence and one-sided dedication, to some extent. However, some of HK patients may be simultaneously more open to have emotional relationship with others. Based on the above, our finding suggests Japanese clinical patients, even though with or without hikikomori condition, may tend to have an immature and undifferentiated desire for dependence and one-sided dedication.

These behavioral patterns have previously been discussed in line with “amae” proposed by Japanese psychoanalyst Takeo Doi (4, 8, 25, 26). The concept of “amae” has been studied as an origin of interdependence between mother and child in Japanese culture, and as a desire to obtain integrated feelings through passive communication with each other (25). The point is that persons with hikikomori may protect themselves by staying in primitive interdependent relationships with significant others (especially mother), even when they come to enter adulthood that requires social engagement as an independent self. Our findings also suggest a psychological aspect of hikikomori as they expect others to presume their feelings and thoughts without their own assertion. This aspect may make it difficult to face and deal directly with problems in social life.

A previous study has suggested difficulty in revealing pathological aspects of dependence such as “amae” in Japanese society (27). Interestingly, there are no significant differences of dependent personality traits between HK patients and non-HK patients using the self-rated questionnaire. The present study suggests CS to be an insightful tool clarifying deeper psychological characteristics and society-based unconscious aspects such as “amae,” which are difficult to assess solely through self-rated scales.

From the above, our findings suggest that hikikomori phenomenon may have the aspect of coping behaviors to satisfy one’s desire for dependence. We assume at least some persons with hikikomori condition have the groundwork of counting on others, when confronting difficulties. Furthermore, tendency to regulate expressing emotions can be understood as one’s ability to try to adapt oneself the environment. However, if a person suppresses his/her feelings and controls emotional expression too much, he/she may easily get exhausted. Especially when he/she can only express his/her anger and aggression indirectly or implicitly, his/her social interaction may become filled with frustration. Difficulty in making the shift from primitive dependence and attachment on significant others may lead to avoiding hardship in social interaction and prolonging the period of hikikomori condition. We propose it is important to notice that not all persons with hikikomori avoid emotional relationship with others. The groundwork of counting on others may be a key to support for hikikomori sufferers.

## Limitation and Future Perspectives

The present study has some limitations. The main limitation is small sample size. Next, participants had a variety of psychiatric disorders and socioeconomic backgrounds. In the present study, we found quite different personality tendencies between HK patients and non-HK patients, by regulating the two groups according to *DSM-IV* axis I diagnoses. However, we did not consider personality tendencies, because of the small sample size. Our findings imply that personality traits are strongly related to hikikomori phenomenon. Future research regulating samples according to SCID-II personality traits is warranted to address the unconscious-level characteristics of hikikomori and non-hikikomori beyond artificial personality traits. The third limitation is the validation process of CS scores. In the present study, the CS administrator (AI) evaluated the tentative CS scores and finally validated by obtaining the consensus with another psychologist (RK). More standardized validation methods should be applied for future research. Finally, we did not use multiple test correction to avoid the risk of false negatives, as the purpose of this study was the inclusive analysis of CS variables and hikikomori to serve exploratory pilot outcomes for future validation (25). We successfully grasped valuable CS variables with statistically significant difference. Since candidate variables related to hikikomori phenomenon have been identified, future validation study, especially focused on CS variables and clusters related to emotional regulation, dependence, and relationships with others, is warranted. Moreover, clinical studies with greater sample size should be conducted to validate our preliminary findings and psychological aspects and hikikomori in more detail.

In conclusion, we propose the psychological characteristics of hikikomori as follows: 1) difficulty in expressing emotions and feelings directly (according to the result of passive aggressive personality trait and large FC), 2) difficulty in becoming independent from relationships based on strong desire for basic affection and emotional support (according to the result of large SumT), and 3) expecting others to presume his/her feelings and thoughts without his/her own assertion (according to the result of FC, SumT, and Fd). Future research should be conducted whether FC, SumT, and Fd in CS could be predictive indicators when evaluating deeper personality aspects of each patient with hikikomori and selecting precision treatments for him/her. Since limited empirical research has been conducted regarding hikikomori, CS has potential for application in clinical research settings with greater sample size, such as comparisons of therapeutic effects among several treatments.

## Data Availability Statement

The datasets generated for this study are available on request to the corresponding author.

## Ethics Statement

This study was carried out in accordance with the recommendations of Ethical Guidelines for Medical and Health Research Involving Human Subjects, published by Japan’s Ministry of Health, Labor, and Welfare, and the ethics committee of Kyushu University with written informed consent from all subjects. All subjects gave written informed consent in accordance with the Declaration of Helsinki. The protocol was approved by the ethics committee of Kyushu University.

## Author Contributions

The corresponding author TK contributed to the conception and design. TK, AI, RK, KK, HK, and NK contributed to the investigation. RK, AI, SI, HK, and TK contributed to the data checking, analysis, and interpretation of data. FF and NK checked the process of interpretation of data and data analysis. RK, AI, and TK drafted the article, and HK, NK, FF, SI, and SK revised it critically for important intellectual content. All the authors provided final approval of the version to be published.

## Funding

This research project is partially supported by Grant-in-Aid for Scientific Research on 1) Innovative Areas “Will-Dynamics” of the Ministry of Education, Culture, Sports, Science and Technology, Japan (JP16H06403 to TK), 2) the Japan Agency for Medical Research and Development (AMED) (Syogaisya-Taisaku-Sogo-Kenkyu-Kaihatsu-Jigyo to TK and SK (JP17dk0307047, JP18dk0307075, and JP19dk0307073), and Yugo-No to TK (JP18dm0107095), and 3) KAKENHI—the Japan Society for the Promotion of Science (JP26713039, JP15K15431, JP16H03741, and JP18H04042 to TK, and JP16H02666 to SK). All the funders had no role in study design, data collection and analysis, decision to publish, or preparation of the manuscript.

## Conflict of Interest Statement

The authors declare that the research was conducted in the absence of any commercial or financial relationships that could be construed as a potential conflict of interest.
